# Romantic Love vs. Drug Addiction May Inspire a New Treatment for Addiction

**DOI:** 10.3389/fpsyg.2016.01436

**Published:** 2016-09-22

**Authors:** Zhiling Zou, Hongwen Song, Yuting Zhang, Xiaochu Zhang

**Affiliations:** ^1^Faculty of Psychology, Southwest UniversityChongqing, China; ^2^Center of Medical Physics and Technology, Hefei Institutes of Physical Science, CASHefei, China; ^3^Chinese Academy of Sciences Key Laboratory of Brain Function and Disease, School of Life Sciences, University of Science and Technology of ChinaHefei, China; ^4^School of Humanities and Social Science, University of Science and Technology of ChinaHefei, China; ^5^Centers for Biomedical Engineering, University of Science and Technology of ChinaHefei, China

**Keywords:** romantic love, social cognition system, drug addiction, oxytocin, resting-state functional connectivity, drug addiction treatment

## Abstract

Drug addiction is a complex neurological dysfunction induced by recurring drug intoxication. Strategies to prevent and treat drug addiction constitute a topic of research interest. Early-stage romantic love is characterized by some characteristics of addiction, which gradually disappear as the love relationship progresses. Therefore, comparison of the concordance and discordance between romantic love and drug addiction may elucidate potential treatments for addiction. This focused review uses the evidences from our recent studies to compare the neural alterations between romantic love and drug addiction, moreover we also compare the behavioral and neurochemical alterations between romantic love and drug addiction. From the behavioral comparisons we find that there are many similarities between the early stage of romantic love and drug addiction, and this stage romantic love is considered as a behavioral addiction, while significant differences exist between the later stage of romantic love and drug addiction, and this stage of romantic love eventually developed into a prosocial behavior. The neuroimaging comparisons suggest that romantic love and drug addiction both display the functional enhancement in reward and emotion regulation network. Except the similar neural changes, romantic love display special function enhancement in social cognition network, while drug addiction display special dysfunction in cognitive control network. The neurochemical comparisons show that there are many similarities in the dopamine (DA) system, while significant differences in oxytocin (OT) system for romantic love and drug addiction. These findings indicate that the functional alterations in reward and emotion regulation network and the DA system may be the neurophysiological basis of romantic love as a behavioral addiction, and the functional alterations in social cognition network and the OT system may be the neurophysiological basis of romantic love as a prosocial behavior. It seems that the OT system is a critical factor for the development of addiction. So we then discuss strategies to treat drug addiction with OT, and suggest that future research should further investigate OT system interventions aiming to improve cognitive control and/or social cognition functions, in order to develop strategies designed to more effectively treat drug addiction.

## Introduction

**Romantic love** can be defined as “a state of intense longing for union with another” (Hatfield and Rapson, 1987). The early stage of romantic love is usually characterized by intense emotional responses such as euphoria, intensely focused attention on the preferred individual, obsessive thought about the person, emotional dependency on and craving for emotional union with the beloved person (Hatfield and Rapson, 1987; Aron et al., 2005).

KEY CONCEPT 1 **Romantic love**

Romantic love is defined as “a state of intense longing for union with another” (Hatfield and Rapson, 1987, page 260).

Some researchers regard romantic love as a type of behavioral addiction (Burkett and Young, 2012; Fisher et al., 2016). People who are in the early stage of romantic love express many similar traits with addicts (Liebowitz, 1983; Hatfield and Sprecher, 1986; Meloy and Fisher, 2005; Association, 2013). They focus on their beloved (salience); they yearn for the beloved (craving); they feel a “rush” of exhilaration when seeing or thinking about their beloved (euphoria/intoxication). As their relationship builds the lover seeks to interact with the beloved more (tolerance). If the beloved breaks off the relationship, the lovers experience the common signs of drug withdrawal, such as lethargy, anxiety, insomnia, or hypersomnia, loss of appetite or binge eating, irritability, and chronic loneliness (Fisher et al., 2016).

Our recent study that used resting state functional magnetic resonance imaging (rsfMRI) to study love-related changes in resting state functional connectivity provided some new and important evidences for romantic love related functional alterations in brain network (Song et al., 2015). Combined our previous studies about romantic love (Wang et al., 2016) and addiction (Zhang et al., 2009, 2011; Lv et al., 2016; Wei et al., 2016), we found that romantic love and addiction have the significant differences in resting-state brain network. Therefore, systematic comparison between romantic love and addiction may be helpful to understand the similarities and differences of them, and may inspire a new possible treatment to addiction.

In general, romantic love usually undergoes two phases. The first phase of romantic love is falling in love which has more a character of excitation and stress (Marazziti and Canale, 2004; Aron et al., 2005). After a few months (about 6 months) the relationship develops into second phase which has a character of calm, safety and balance (Stárka, 2007).

For the development of addiction, researchers suggest that the neuroplasticity of addiction consists of two phases, too. During initiation drug use is completely deliberate and voluntary (regulated relapse), and the transient changes in neural functioning continued for hours up to weeks during abstinence (Kalivas and O'Brien, 2008). While during the second phase, drug use is compulsive, and the changes in neuroplasticity become “stable” which last for weeks up to permanency, and turn to be fundamental for the maintenance of the addiction, and are responsible for relapse after a period of abstinence (Kalivas and O'Brien, 2008).

From the perspective of the development stage of romantic love, the early stage of romantic love shows more addictive characteristics. Therefore, the early stage of romantic love is often regarded as a type of behavioral addiction. Although romantic love and **drug addiction** are similar in the early stages, they are different in subsequent stages, as the addictive characteristics of love gradually disappear as the romantic relationships progresses. However, the addictive characteristics are gradually magnified with repeated use of drugs of abuse. Finally, romantic love and drug addiction develop into different behaviors and have different effects on human development.

KEY CONCEPT 2 **Drug addiction**

Drug addiction is a state of compulsive drug use; despite treatment and other attempts to control drug use, addiction is likely to persist.

As romantic love displays some addictive behaviors, and there are many concordances and discordances in development progression of romantic love and drug addiction. Therefore, in this article our hypothesis are that: romantic love and drug addiction may have many concordances and discordances in brain cortical functioning and neuroendocrine factors; and (1) these concordances may be the neurophysiological basis of romantic love as a behavioral addiction, and (2) these discordances may be the neurophysiological basis of romantic love as a prosocial behavior; and (3) these discordances may be the critical factors for the development of drug addiction and further study about these critical factors may help to find a new treatment for drug addiction. In order to verify our hypothesis, we compare romantic love and drug addiction from three relevant aspects: behavioral characteristics, brain functioning and neuroendocrine factors.

The comparisons between romantic love and drug addiction indicate that romantic love and drug addiction both display the functional enhancement in reward and emotion regulation network. Except the similar neural changes, romantic love display special function enhancement in social cognition network, while drug addiction display special dysfunction in cognitive control network. The comparisons of neurochemical alterations show that there are many similarities in DA system, while significant differences in OT system for romantic love and drug addiction. These indicate that the functional alterations in reward and emotion regulation network and the DA system may be the neurophysiological basis of romantic love as a behavioral addiction, and the functional alterations in social cognition network and the OT system may be the neurophysiological basis of romantic love as a prosocial behavior. It seems that the OT system is a critical factor for the development of addiction. Further, study the OT system may help to find a new treatment for drug addiction.

## Comparisons of romantic love and drug addiction: the behavioral characteristics

Fisher (1998) proposed that mammals typically exhibit three primary categories of mating- and reproduction-related emotions: lust, attraction, and attachment. Lust is characterized by cravings for sexual gratification, and it motivates individuals to seek sexual union with specific individuals. Attraction is characterized by increased energy and focused attention in mammals, thereby facilitating mate choice and enabling individuals to focus on a partner. Attachment is characterized by maintenance of close social contact in mammals, and is described as feelings of calm, comfort, and emotional union between human partners. Lust and attraction are the primary emotional categories that correspond to the core characteristic “longing for union” during early romantic love. This core characteristic is very similar to addictive symptoms, including the induction of intense drug-seeking behavior by either the drug itself or drug-related cues. Additionally, the negative emotions induced by love disruption are also similar to the acute withdrawal symptoms following drug discontinuation. From the perspective of considering love to be an attachment process, romantic love and drug addiction may have additional similarities. Furthermore, DSM-5 criteria describe some more detailed comparisons between drug addiction and romantic love (see Table 1).

**Table 1:** **DSM-5 criteria and other characteristics of substance use disorders as compared to love**.

**Substance use disorders criteria and other symptoms**	**Similarities in love**	**Differences in love**
**IMPAIRED CONTROL**		
Substance is taken in larger amounts or over a longer period than originally intended	Sensation of “time flying” when with the partner	
Persistent desire or repeated to cut down or regulate substance use and unsuccessful attempt to decrease or discontinue use	Sensation of not being able to stay away from the partner; failed attempt (s) to break up	
Spending a great deal of time to obtain, use, recover	Dating	
The craving for drug easily induced by drug and drug-associated cues	The longing for reciprocity easily induced by partner and partner-associated stimuli	
**SOCIAL IMPAIRMENT**		
Result in a failure to fulfill major role obligations at work, school, or home		The romantic relationship may improve the social cognition
Continued substance use despite having persistent or recurrent social or interpersonal problems caused or exacerbated by the effects of the substance		Do not cause social or interpersonal problems
Important social, occupational, or recreational activities are given up or reduced	Less of time with friends	
**RISKY USE OF THE SUBSTANCE**		
Continued use despite knowledge of a persistent or recurrent physical or psychological problem that is likely to have been caused or exacerbated by use	(a very few case) Physically or emotionally abusive relationships; staying with someone who “isn't right for you”	In most cases, especially female, when clear know someone who “isn't right for you,” individual will end the relationship
**PHARMACOLOGICAL CRITERIA**		
Tolerance (marked increase in amount; marked decrease in effect)	Transition from early euphoria to contentment	
Characteristic withdrawal symptoms; substance taken to relieve withdrawal	Grief (from loss); separation anxiety when apart	
**OTHER SYMPTOMS**		
Stress-induced reinstatement	Consolation-seeking	
Drug and drug-associated stimuli induce the intense physiological arousal	In early romantic love, partner and partner-associated stimuli induce the intense physiological arousal	A few months after initially fall in love, the intense physiological arousal gradually reduce
Chronic drug abuse induce the impairment of decision-making		Individuals display a better decision-making, such as commitment, to establish the healthy long-term relationship
Compulsive drug-seeking behavior; obsessive thinking for drug	In early romantic love, intrusive thinking or preoccupation with the partner; obsessive thinking for partner	A few months after initially fall in love, the obsessive thinking gradually reduce and is replaced by calm, safety and balance
Chronic drug abuse induce the impairment of inhibitory control; higher impulsivity		Lovers do not display the impairment of inhibitory control and the changes of impulsivity

While romantic love is rarely considered a pathological disorder (Burkett and Young, 2012), early phase romantic love (“falling in love”) is characterized by euphoria, intensely focused attention on the preferred individual, obsessive thinking about the person, increased energy, as well as emotional dependency on and craving for emotional union with the beloved (Aron et al., 2005). The early phase of romantic love lasts ~6 months (Marazziti and Canale, 2004). After a few months, the stress-liking features, such as obsessive thinking, emotional dependency, and craving, subside and are replaced by feelings of calm, safety, and balance (Stárka, 2007). Additionally, for the long-term relationship, the feelings of closeness and the decision making about the commitment to a relationship play relatively large parts (Sternberg, 1986).

However, for people with drug addiction, behaviors progress from initial self-administration to impulsive drug use, and finally develop into compulsive drug use. With repeated drug use, the stress-liking features, and the detrimental effects become progressively more serious. Moreover, for the repeated drug abuse, the obsessive drug-seeking and craving for abused drug play the key roles (Volkow et al., 2006).

Generally, in the early stage of romantic love, lovers display some addictive characteristics, primarily the stress-liking features. However, the stress-liking features changes differently over time in people with drug addiction, becoming progressively worse with repeated drug use. Understanding these differences may contribute to discovering treatments for preventing and curing addiction.

## Comparison of romantic love and drug addiction: brain functioning

Comparison of neuroimaging studies regarding romantic love and drug addiction must consider two primary outcomes: (a) the response to direct effects of the partner or drug-related cues, and (b) spontaneous neural activity without effects induced by known external stimulation. Responses to directly related cues reflect state neural activity and spontaneous neural activity primarily reflects trait neural activity (Fox and Raichle, 2007).

### Exposure to partner or drug-related cues

When viewing pictures of their partner, lovers show significant activation in some brain regions which include the ventral tegmental area (VTA), nucleus accumbens (NAC), caudate, insula, dorsal anterior cingulate cortex (dACC), dorsolateral prefrontal cortex (dlPFC), hippocampus, posterior cingulate cortex (PCC), precuneus, temporo-parietal junction (TPJ), and hypothalamus (Bartels and Zeki, 2000; Aron et al., 2005; Ortigue et al., 2007; Xu et al., 2011; Acevedo et al., 2012). In our recent study, we found that the NAC and medial prefrontal cortex (mPFC) show activation while lovers were exposed to their partner related cue (Wang et al., 2016).

For addicts, while exposed to drug cues, they show significant activation in many brain regions which include the VTA, NAC, caudate, insula, dACC, dlPFC, mPFC, ventral anterior cingulate cortex (vACC), medial orbitofrontal cortex (mOFC), inferior frontal gyrus (IFG), and amygdale (Volkow et al., 2003; Baler and Volkow, 2006). Moreover, our earlier study found that when exposed to smoke-related cue the dlPFC, mPFC, vACC, occipital cortex, insula, right amygdala and dACC showed significant activity in smokers (Zhang et al., 2009, 2011). More recently, we reviewed the addiction-related cue-induced neural changes, addicts showed that right orbitofrontal cortex (OFC), right NAC, bilateral anterior cingulated (ACC), mPFC, right dlPFC, right caudate nucleus, left parahippocampus ware activated by addiction-related cues (Zhang et al., 2016).

**Figure 1:**
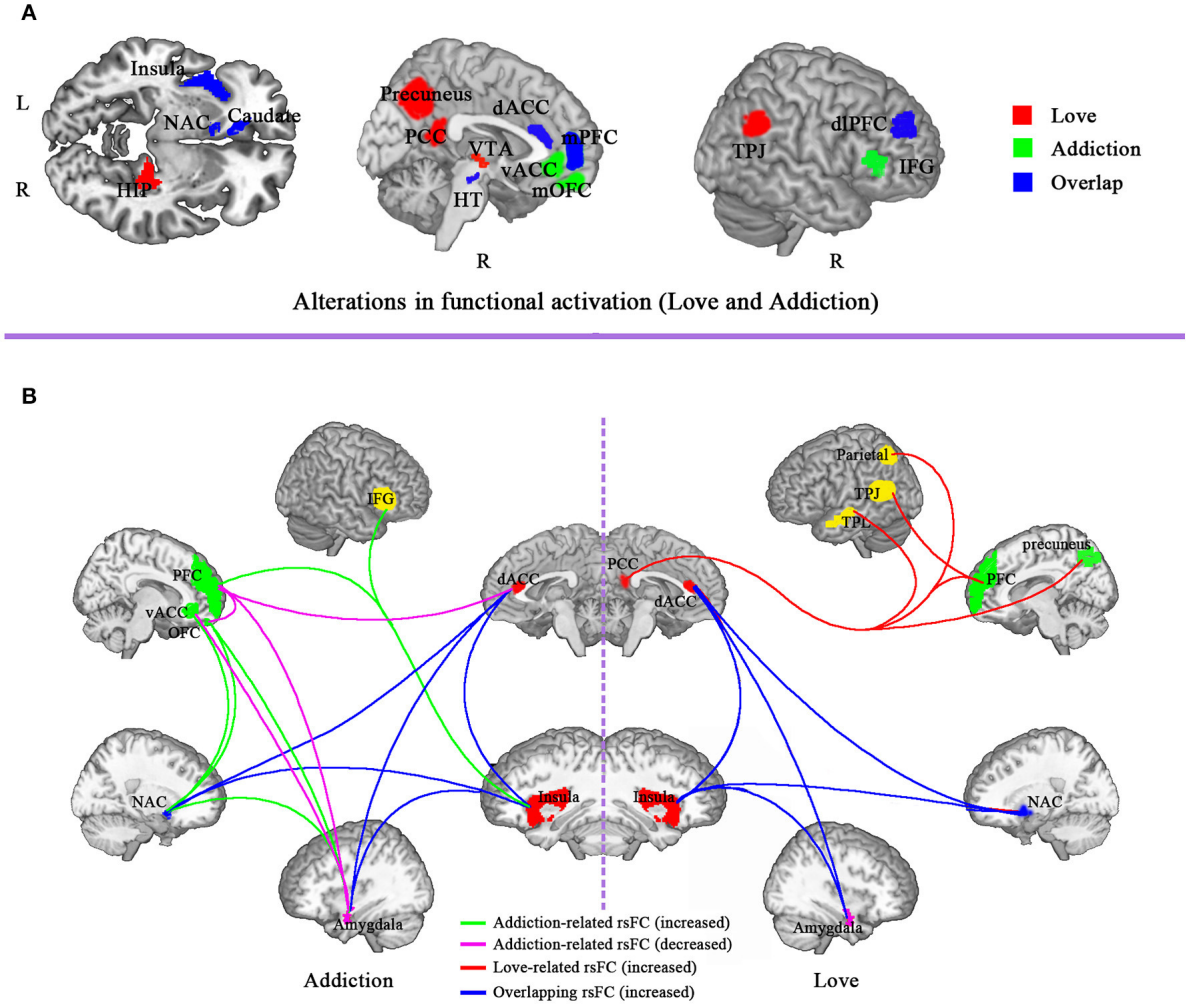
**Comparison of brain functional alteration between drug addiction and romantic love. (A)** Alterations in functional activation in brain regions involved in love and addiction. Red, love-related functional activation; green, addiction-related functional activation; blue, overlap of functional activation for love and addiction. **(B)** Alterations in resting-state functional connectivity (rsFC) for love and addiction. Green, addiction-related functional connectivity increase; Red, love-related functional connectivity increase; blue, overlap functional connectivity increase for love and addiction; purple, addiction-related functional connectivity decrease. ACC, anterior cingulated cortex; vACC, ventral anterior cingulate; dACC, dorsal anterior cingulate cortex; PCC, posterior cingulate cortex; OFC, orbitofrontal cortex; IFG, inferior frontal gyrus; dlPFC, dorsolateral prefrontal cortex; PFC, prefrontal cortex; VTA, ventral tegmental area; NAC, nucleus accumbens; TPJ, temporo-parietal junction, HIP, hippocampus; HT, hypothalamus; TEP, temporal cortex.

It is obvious that the brain regions of romantic love overlap with drug addiction include VTA, NAC, caudate, insula, dACC, mPFC, and dlPFC (see Figure 1). Other than the overlapping regions, there are additional brain regions that are activated only during romantic love or drug addiction (see Figure 1). Specifically, activated brain regions for romantic love include the hippocampus, PCC, precuneus, TPJ, and hypothalamus. Regions uniquely activated in drug addicts include the mPFC, vACC, mOFC, IFG, and amygdala.

The VTA, NAC, and caudate comprise the mesolimbic system, and are primarily associated with pleasure, general arousal, focused attention and motivation to pursue and acquire rewards and mediated primarily by dopamine system activity (Delgado et al., 2000; Schultz, 2000; Elliott et al., 2003). These regions of the reward system are directly associated with addiction in many studies of addiction (Breiter et al., 1997; Panksepp et al., 2002; Melis et al., 2005; Volkow et al., 2007; Frascella et al., 2010) and romantic love (Bartels and Zeki, 2000; Aron et al., 2005; Ortigue et al., 2007; Xu et al., 2011; Acevedo et al., 2012).

The dACC contributes to monitoring conflict via information processing, and facilitates compensatory adjustments to cognitive control (Botvinick et al., 2004). The PCC contributes to social cognition, and social trustworthiness (Maddock, 1999; Winston et al., 2002). During romantic love, increased activation in the dACC is associated with suppressed obsessive thinking (Aron et al., 2005), and altered activation in the PCC indicates that social cognition and social trustworthiness significantly increase with increased duration of the love relationship. For example, when viewing pictures of a romantic partner, activation of the dACC and PCC indicates a significant positive correlation with the duration of the love relationship. Additionally, scatter plots of dACC or PCC activity and duration of the relationship indicate that lovers in longer relationships (8–17 months) respond differently than those in relatively short relationships (1–7 months). Furthermore, the dACC and the PCC are negatively activated during short relationships (1–7 months), but positively activated during longer relationships (8–17 months) (Aron et al., 2005). The addictive characteristics (e.g., stress-liking) disappear ~6 months after initially falling in love, therefore, alterations in dACC and PCC activation are potential neural bases for the disappearance of addictive characteristics.

Activation of the ventral prefrontal cortex, especially the mPFC, mOFC, and vACC, is correlated with the strength of cravings in people with drug addiction (Goldstein and Volkow, 2011). However, activation of the dorsal prefrontal cortex, especially the dlPFC, dACC, and IFG, is correlated with suppression of drug cravings. For example, instructions to resist cravings while viewing smoking-related videos resulted in increased activity in the dlPFC, dACC, and IFG in smokers, as well as decreased activity in the mOFC and vACC (Goldstein and Volkow, 2011).

**Figure 2:**
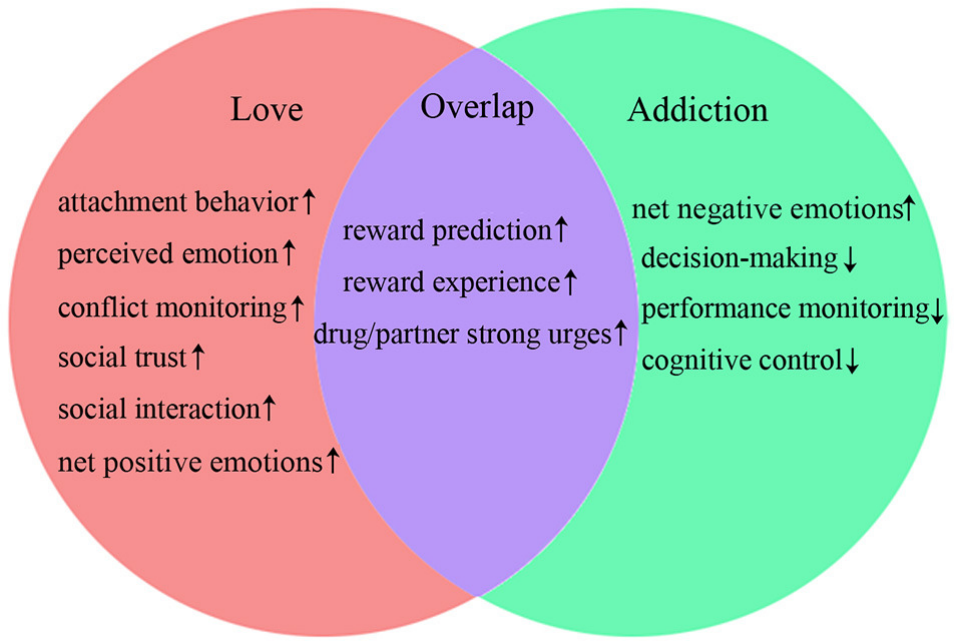
**Comparison of cognitive functions**. ↑, function increases; ↓, function decreases.

Taken together, the overlapping brain regions activity during romantic love and drug addiction indicate that both lovers and people with addiction display activated basic stimulus detection functions (such as reward prediction and reward experience), as well as activated primary emotional reactions (strong urges for drug/partner) when exposed to partner-or drug-related cues (see Figure 2). However, romantic love and drug addiction can be differentiated based on other cognitive functions (see Figure 2). People with addiction experience strong drug cravings and obsessive thinking. However, in lovers, the obsessive thinking and stress-liking features gradually disappear, and conflict monitoring and social cognition are gradually enhanced as the love relationship progresses, and the dACC and PCC may be involved in these changes. Additionally, the neural changes may alter the relationship progression, and the love relationship eventually does not develop into addiction.

### Spontaneous neural activation during love and addiction

In this section, we will use the evidences that we have provided previously to compare the romantic love related and addiction-related resting-state functional connectivity (rsFC). rsFC is an important index for measuring spontaneous neural activity, reflecting functional communication between brain regions. In general, rsFC is defined as the temporal dependency between spatially remote neurophysiological events (Van Den Heuvel and Pol, 2010). Compared with task-activation rsFC has a better signal to noise ratio, and can be used to study multiple cortical systems (Fox and Greicius, 2010). Most of the previous neuroimaging studies of romantic love only focused on the specific external stimuli induced functional changes of brain regions (Bartels and Zeki, 2000; Aron et al., 2005; Ortigue et al., 2007; Xu et al., 2011; Acevedo et al., 2012), and did not provide evidences about romantic love related functional alteration of brain network. In our previous original research study (Song et al., 2015), which was published on *frontiers in human neuroscience*, we used resting state functional magnetic resonance imaging (fMRI) to study love-related changes in resting state functional connectivity. Our study provided new and important evidences of romantic love related functional alteration of brain network, which suggested that the love-related functional alterations was also observed at the brain network level.

Specifically, in our previous original research study (Song et al., 2015), we used rsfMRI data to compare the functional connectivity (FC) across an “in-love” group (LG, currently intensely in love), an “ended-love” group (ELG, ended romantic relationship recently), and a “single” group (SG, never fallen in love). Results showed that rsFC within the reward and emotion regulation network (dACC, insula, caudate, amygdala, and NAC) as well as rsFC in the social cognition network (TPJ, PCC, MPFC, inferior parietal, precuneus, and temporal lobe) was significantly increased in the LG (in comparison to the ELG and SG).

The addiction related studies showed that addicts displayed increased rsFC within the reward and emotion regulation network (dACC, insula, amygdala, and NAC); between the reward circuit and the motivation circuit (NAC-vACC, NAC-OFC), as well as between the memory/learning circuit and the motivation circuit (amygdala-OFC; Gu et al., 2010; Ma et al., 2010; Sutherland et al., 2012). Moreover, our previous studies showed that rsFC between mPFC and insula, and between dACC and insual displayed significant increased in smoker (Zhang et al., 2011; Wei et al., 2016).

It is obvious that the increased rsFC of romantic love overlaps with that of drug addiction include rsFC between the insula and dACC, between the insula and NAC, between dACC and NAC, between dACC and amygdala, and between the insula and amgdala. While other rsFC indices are specific to love or addiction (Gu et al., 2010; Ma et al., 2010; Sutherland et al., 2012; Song et al., 2015; see Figure 1).

The ACC, NAC, insula, and amygdala are core components of the brain systems that play an important role in the processing of sensory and emotional information, reward, and motivational processes (Mogenson et al., 1980). These increased rsFC indicated that the reward signal processed in NAC and the emotion signals processed in amygdala are transmitted to the insula more effectively. The control of a goal-directed behavior will involve both the insula, representing awareness, and the ACC, representing the control of directed effort (Craig, 2009). Thus, these increased rsFC are consistent with more integration of conscious awareness within conflict monitoring processes, and may be responsible for promoting individuals to detect drug- or partner-related cues with greater sensitivity. Moreover, this may be the neural basis of incentive-sensitization theory of addiction, which suggested that repeated exposure to potentially addictive drugs can persistently change brain cells and circuits that normally regulate the attribution of incentive salience to stimuli, a psychological process involved in motivated behavior, and the nature of these “neuroadaptations” is to render these brain circuits hypersensitive in a way that results in pathological levels of incentive salience being attributed to drugs and drug-related cues (Robinson and Berridge, 1993).

Similarly to task-related activation of brain regions, overlapping rsFC demonstrates that both lovers and people with addiction experience activated basic conscious awareness and more integration of partner- or drug-related conscious awareness within conflict monitoring processes (see Figure 2). Furthermore, lovers demonstrate more effective awareness of emotional states, enhanced conflict monitoring, increased social trustworthiness, and improved social interaction abilities. These skills may mitigate stress-liking features, thereby facilitating progression of the romantic relationship.

Except for the overlapping, lovers display specifically increased rsFC within the social cognition network (e.g., the temporo-parietal junction (TPJ), PCC, medial prefrontal cortex (mPFC), inferior parietal cortex, precuneus, and temporal lobe; Song et al., 2015). These alterations in functional connectivity may indicate that lovers have more social trust, and have better social interaction abilities. These skills may mitigate stress-liking features and contribute to maintaining the romantic relationship.

Except for the overlapping, people with addiction display specifically increased rsFC between the reward circuit and the motivation circuit (NAC-vACC, NAC-OFC), as well as between the memory/learning circuit and the motivation circuit (amygdala-OFC). Specifically, decreased rsFC indices are observed between the control circuit and the motivation circuit (PFC-OFC), between the cognitive circuit and the memory/learning circuit (PFC-amygdala, and vACC-amygdala), and within the control circuit (PFC-ACC; Gu et al., 2010; Ma et al., 2010). These alterations in functional connectivity support the addiction model proposed by Volkow et al. (2003), and indicate enhanced salience for an addictive drug and its related cues, combined with reduced cognitive control during addiction (Ma et al., 2010). The rsFC between the insula and the control circuit is also altered in people with addiction. Specifically, the ventral and dorsal anterior insula have significantly increased functional connectivity with the dACC, dorsal mPFC, and lateral PFC. Furthermore, the posterior insula displays significantly decreased functional connectivity with the dACC, dorsal mPFC, lateral PFC, and anterior insula (Cisler et al., 2013). Posterior insula cortex activity is associated with changes in bodily states, and the anterior insula cortex mediates subjective awareness of bodily states. Increased functional connectivity between the anterior insula and prefrontal networks indicates enhanced awareness or detection of drug cues or internal craving-related bodily states, and may contribute to biased decision-making processes. Decreased functional connectivity between the posterior insula and prefrontal networks, as well as between the posterior insula and anterior insula, indicate that the influence of somatic states on performance monitoring and attention is diminished (Cisler et al., 2013).

These different alterations of rsFC indicate that people with addiction also experience reduced cognitive control, performance monitoring lacking regulation of the somatic states, and over-valuation of the salience of the drug and its related cues. These alterations may result in subsequent impulsive and compulsive repeated drug consumption, or relapse after protracted periods of abstinence. In general, the results of comparisons of rsFC between romantic love and drug addiction display some similarities to the results of comparisons of task-induced brain activation. Although there are some differences between romantic love and drug addiction (see Figure 2), the reduced cognitive control and the weakened social cognition may be the most important differences between romantic love and drug addiction. Developing strategies to improve cognitive control and social cognition may therefore be useful for treating drug addiction.

## Comparison of romantic love and drug addiction: neuroendocrine factors

Pair bonding and romantic love have previously been conceptualized as attachment processes (Hazan and Shaver, 1987; Fraley and Shaver, 2000). In order to distinguish animal and human studies, we describe this process as pair bonding in animals and as romantic love in humans. However, most neuroendocrine studies of pair bonding and drug addiction are conducted in animals. Therefore, we compare neuroendocrine alterations between pair bonding and drug addiction (see Table 2) by reviewing several animal models. Neurotransmitters and neuropeptides involved in pair bonding and drug addiction include primarily dopamine (DA), corticotropin-releasing factor (CRF), oxytocin (OT), and arginine vasopressin (AVP). DA, CRF, OT, AVP, and their receptors (including DA receptors: D1R and D2R, CRF receptors: CRF-R1 and CRF-R1, the OT receptor: OTR, and AVP receptors:V1aR, V1bR, and V2R) contribute significantly to pair bonding and drug addiction. DA is generally regarded as a crucial contributor to the biological reward and motivational processes (Esch and Stefano, 2005). CRF is involved in the neurobiology underlying stress, fear, and anxiety (Bale and Vale, 2004). Additionally, OT and AVP are associated with processing and retention of social information (Hollander et al., 2007).

**Table 2:** **Function and alterations of neurochemical systems involved in love and addiction**.

	**Love**	**Drug addiction**
**MAINTENANCE**		
DA	D1R promotes maintenance	D1R and D2R promote maintenance
	Plasticity in striatalD1R promotes maintenance	Plasticity in striatal D2R promotes maintenance
CRF	CRF promotes maintenance	CRF-R1 promotes maintenance
		CRF-R2 may inhibit maintenance
	Plasticity in CRF promotes maintenance	Plasticity in CRF promotes maintenance
OT	OT is not necessary for maintenance	OTR inhibits maintenance
		Plasticity in the OT system promotes maintenance
**DISRUPTION**		
DA		D2R promotes relapse
		
CRF	Released after disruption	Released after disruption
	Plasticity in hypothalamic CRF promotes return to partner	Plasticity in hypothalamic CRF-R1 promotes relapse, CRF-R2 may inhibit relapse
		
OT	Released after disruption	Released after disruption
	OT inhibits return to partner	Endogenous plasticity in the OT system promotes relapse
		Exogenous OT inhibits relapse

*DA, dopamine; CRF, corticotropin-releasing factor; OT, oxytocin; AVP, arginine vasopressin*.

In this section, neurochemical alterations during both maintenance and disruption of pair bonding and drug addiction will be discussed.

### Maintenance of pair bonding and drug addiction

Neurotransmitters and neuropeptides are important contributors to the maintenance of both pair bonding and drug addiction. Activation of D1R promotes maintenance of pair bonding and drug abuse, and CRF increases in the NAC also promote pair bonding maintenance (Aragona et al., 2006; Grippo et al., 2007; Burkett and Young, 2012). Additionally, D2R and CRF-R1 activation in the central nervous system also promote maintenance of drug use; however, activation of OTR and CRF-R2 inhibit drug addiction maintenance (Carson et al., 2010; Koob, 2010; Zanos et al., 2014). Pair bonding and chronic exposure to drugs of abuse induce plasticity in DA and CRF receptors, which promotes the maintenances of pair bonding and drug addiction (Nakajima and McKenzie, 1986; Zorrilla et al., 2001; Bosch et al., 2009; Burkett and Young, 2012).

However, there are also some significant differences between neuroendocrine factors that facilitate the maintenance of pair bonding and drug addiction. Chronic administration of an abused drug produces a decrease in D2R in the NAC (Grieder et al., 2012), as well as decreases in endogenous OT and OTR density in the central nervous system (Johns et al., 1997; Light et al., 2004; Jarrett et al., 2006). These changes in D2R expression in the NAC are associated with decreased baseline metabolic activity in the OFC, ACC, and dlPFC, and these changes may promote compulsive drug intake and reinstatement of drug-seeking behavior (Narendran et al., 2005; Volkow et al., 2008; Asensio et al., 2010). Alterations in endogenous OT indicate that the endogenous OT system may be not sufficient to affect drug-induced consequences, although the exogenous OT is a potential treatment for drug addiction. Similar changes do not occur during pair bonding. As pair bonding continues, D2R do not display plastic changes combined with functional enhancement of the OT system (Burkett and Young, 2012; Schneiderman et al., 2012). These plastic changes are potential neural mechanisms underlying the differences between the development of addiction and love. As described above, lovers display addictive characteristics during early romantic love, but these characteristics gradually disappear as the romantic relationship progresses. Functional changes to the OT system may be the neural basis of this process. Other than reduced functioning of the OT system, people with addiction also experience D2R functional damage and decreased baseline metabolic activity in the OFC, ACC, and dlPFC. These findings suggest that stress-liking features become more intense, and also that cognitive control is impaired with repeated drug abuse.

### Disruption of pair bonding and drug addiction

Similarly to drug addiction withdrawal, a disruption to pair bonding often induces profound grieving, anxiety, stress, and depressive-like behaviors (Bosch et al., 2009). In conjunction with these symptoms, CRF and OT increase significantly in the central nervous system (Bartz and Hollander, 2006; Grippo et al., 2007). Furthermore, disruption of pair bonding induces plastic changes in the hypothalamic CRF system, and these changes are associated with individuals returning to their former partner (Grippo et al., 2007). Although disruption of drug use also induces plastic changes in the hypothalamic CRF system, the changes to CRF-R1 and CRF-R2 have different effects on subsequent relapse, with promoting and inhibiting effects, respectively (Koob, 1999). OT has similar effects on the disruption of both pair bonding and drug use, including inhibiting return to the previous partner or addiction relapse. This effect likely occurs via relieving disruption-induced anxiety, stress, and depressive-like behaviors (Kovács et al., 1981; Gibbs, 1984; Cui et al., 2001; Pedersen et al., 2013). During drug addiction, however, the endogenous OT system may be not sufficient to affect drug-induced consequences, as chronic administration of the abused drug results in lower endogenous OT baseline activity (Light et al., 2004). Therefore, the combination of reduced activity of the endogenous OT system and a dysfunctional prefrontal cortex, described in the previous section, contribute to a high risk for relapse even after protracted periods of abstinence. During pair bonding, functional enhancement of the OT system facilitates maintenance of the relationship, whereas after disruption of pair bonding, functional enhancement of the OT system inhibits return to the partner. These findings indicate that individuals who have experienced love demonstrate improved social adaptation abilities, and enhanced function of the OT system may be the neural basis for this strength. In general, differences occur between romantic love and drug addiction in the maintenance and disruption stages, and changes to the OT system may be the neural basis of these differences.

## A new possible treatment to addiction based on the oxytocin

As described above, comparison of romantic love and drug addiction progression indicates that there are some similarities between the early stage romantic love and drug addiction, and romantic love are considered as a behavioral addiction, while there are many significant differences between the later stage romantic love and drug addiction, and romantic love eventually developed into a prosocial behavior. Moreover, comparison of romantic love and drug addiction using neuroimaging studies indicates that the function enhancement of reward and emotion regulate system may be the neural basis of the similarities between the early stage of romantic love and drug addiction. However, the difference of functional alteration of social cognition system and the cognitive control related brain regions may be the neural basis of the differences between the later stage romantic love and drug addiction. e.g., addicts display the impairment of cognitive control and social cognition. Furthermore, romantic love and chronic administration of abused drug both induce the plastic changes of the DA and OT systems. The previous studies suggested that functional changes in DA system were associated with the dysfunction in reward network (Henry and White, 1995; Aragona et al., 2006; Anderson et al., 2008; Bertran-Gonzalez et al., 2008) and emotion regulation network (Davidson et al., 2000). Moreover, previous study suggested that oxytocin could induce functional connectivity changes within social cognition network (Riem et al., 2013). It seems that the dysfunction of OT system may be the neurochemical basis of the impairment of cognitive control and social cognition in addicts. Therefore, to study how to improve abused drug induced the impairment of the OT system, cognitive control, and social cognition may be help to find the treatment of drug addiction.

Exogenous administration of OT can attenuate the development of tolerance for drugs of abuse, as well as mitigate withdrawal symptoms and minimize reinstatement of drug use (Kovács et al., 1998). For example, repeated administration of cocaine produces behavioral tolerance to the sniffing-induced effects of cocaine. OT pretreatment in cocaine-tolerant rats reduced tolerance for cocaine and produced effects similar to those in non-tolerant control rats (Sarnyai et al., 1992). Additionally, exogenous administration of OT, such as by intranasal administration, increases activity in brain regions associated with cognitive control and social cognition, including the striatum, middle frontal gyrus, dlPFC, IFG, mPFC, OFC, ACC, PCC, and superior temporal sulcus (Riem et al., 2011; Bethlehem et al., 2013; Gordon et al., 2013; Liu et al., 2015). Furthermore, functional connectivity between brain regions (between the amygdala and ACC, between the amygdala and anterior insula) is also enhanced (Bethlehem et al., 2013). These findings indicate that exogenous administration of OT may improve or reverse addictive drug use by reversing D2R-related decreases in baseline metabolic activity in the OFC and ACC, and may partially improve the abnormal functional connectivity between brain regions that occurs during addiction. A recent study suggests that there are also D2R-OTR heteromers with facilitatory receptor–receptor interactions in the striatum, and that these heteromers are associated with changes in social and emotional behavior (Romero-Fernandez et al., 2013). These functional improvements may facilitate cognitive control and performance monitoring in people with addiction, decrease impulsive and compulsive repeated drug consumption, and reduce relapse rates after protracted periods of abstinence. Additionally, the improvement of social cognition by exogenous administration of OT can break the object-orientated rewards and execution of habitual behavioral loops to mitigate the compulsive repeated drug consumption (McGregor and Bowen, 2012).

Although some researchers regard OT administration as a potential treatment for drug addiction (McGregor and Bowen, 2012; Tops et al., 2014), most studies are currently in the preclinical stage (McGregor and Bowen, 2012). Additionally, the mechanisms of OT effects have not yet been elucidated. Through comparing romantic love and drug addiction, we discovered a potential mechanism for the ability of OT to mitigate or reverse the development of drug addiction. These two mechanisms include: (a) mitigation of stress and anxiety, (b) facilitation of social interactions, and (c) the improvement of cognitive control. Activation of stress systems is critical to motivating drug-seeking, which may then develop into drug addiction (Koob, 2008). OT exerts an anti-stress effect and attenuates the stress and anxiety responses (Neumann and Landgraf, 2012). For example, exogenous administration of OT reduces stress-induced corticosterone release and also reduces adrenocorticotropic hormone and CRF mRNA expression (Windle et al., 1997, 2004; Parker et al., 2005). Social interaction may influence drug addiction, and a recent study proposes that activation of OT by facilitating social interaction may attenuate drug addiction (McGregor and Bowen, 2012). Repeated administration of abused drugs induces neuroplasticity of the DA and OT systems that may bias behavior toward object-orientated rewards and execution of habitual behavioral loops (Everitt and Robbins, 2013). Social attachment and exogenous administration of OT may reverse this bias toward social stimuli, which may be associated with termination of object-orientated behaviors and attenuation of the compulsive drug-seeking behaviors (McGregor and Bowen, 2012; Tops et al., 2014). For drug addiction, the tolerance, compulsive drug-seeking, withdrawal symptoms and relapse are the core symptoms. To attenuate the development of tolerance of abused drug and mitigate the withdrawal symptoms may attenuate the development of addiction or partially reverse the the corrosive effects of long-term drugs abuse. However, the relapse and compulsive drug-seeking are the most critical obstacle to treat drug addiction, and the improvement of cognitive control and social attachment may be the key for overcoming relapse and mitigate compulsive drug-seeking (Amaro et al., 1999; Volkow et al., 2006). Exogenous administration of OT can effectively improve the function of cognitive control and social cognition related brain regions and this imply that improving cognitive control and social cognition may be the most important reasons for OT as a potential treatment of drug addiction.

## Summary and future directions

In early romantic relationships, lovers display some characteristics of addiction, but these characteristics disappear after several months. Alterations of functioning in brain regions (especially the dACC and PCC) and neuroendocrine activities (especially the OT system) may be the potential neural bases for the changes in associated behaviors and emotions. During drug addiction, the relapse and compulsive drug-seeking are the most critical obstacle to treat drug addiction (Amaro et al., 1999), and the drug craving is a key contributor to relapse (Volkow et al., 2006). When resisting cravings while viewing drug-related cues, people with addiction experienced increased activity in the dlPFC, dACC, and IFG (Goldstein and Volkow, 2011). These brain regions are primarily involved in cognitive control, and the dACC is critical to monitoring control (Botvinick et al., 2004). The IFG is primarily involved in inhibition and attentional control (Hampshire et al., 2010), whereas the dlPFC contributes to executive control (Wagner et al., 2001). Previous studies suggest that exogenous administration of OT increases activity in these regions (Riem et al., 2011; Gordon et al., 2013; Liu et al., 2015). Exogenous administration of OT can also increase the activity of PCC that is mainly involving in social cognition (Gordon et al., 2013). These findings imply that exogenous administration of OT can improve the cognitive control function and social cognition to inhibit the drug craving and compulsive drug-seeking.

Additionally, we do not compare the brain structure of romantic love and drug addiction. The reason is lack of study that investigates the brain structure of romantic relationship. For romantic love we also do not discuss the resting state brain functioning changes in long period of romantic relationship (the length of relationship more than 18 months). Future research should investigate these questions in order to learn more about the neural mechanism of romantic love.

Most studies addressing treatment of addiction with OT emphasize the effects of OT on attenuating the development of tolerance, as well as on mitigating the withdrawal symptoms of addiction. However, these studies do not comprehensively discuss the therapeutic effects of OT as an addiction treatment. We suggest that OT administration may improve cognitive control in people with drug addiction, thereby inhibiting drug cravings and reducing the probability of relapse. The OT administration can also improve social cognition in people with drug addiction, thereby breaking the object-orientated rewards and execution of habitual behavioral loops to mitigate the compulsive drug-seeking. Therefore, future research should further investigate this possibility and elucidate the mechanisms for OT as a potential treatment for drug addiction.

## Author contributions

Conceive and writing frame design: HS and XZ. Wrote the paper: ZZ and HS. Revise the manuscript: HS, XZ, YZ, and ZZ.

### Conflict of interest statement

The authors declare that the research was conducted in the absence of any commercial or financial relationships that could be construed as a potential conflict of interest.
